# Precise measurement of hyperfine structure in the 3S_1/2_ state of ^7^Li

**DOI:** 10.1038/s41598-017-13531-9

**Published:** 2017-10-16

**Authors:** Pushpander Kumar, Vasant Natarajan

**Affiliations:** Department of Physics, Indian Institute of Science, Bangalore, 560012 India

## Abstract

We report a precise measurement of hyperfine structure in the 3S_1/2_ state of the odd isotope of Li, namely ^7^Li. The state is excited from the ground 2S_1/2_ state (which has the same parity) using two single-photon transitions via the intermediate 2P_3/2_ state. The value of the hyperfine constant we measure is A = 93.095(52) MHz, which resolves two discrepant values reported in the literature measured using other techniques. Our value is also consistent with theoretical calculations.

## Introduction

The three-electron structure of Li lends itself to theoretical calculations with high accuracy, which can be used to test fundamental atomic theories of few-electron systems^1^. However, experimental measurements are complicated by the fact that Li is highly reactive with most transparent materials. This precludes the use of vapor cells (as in the case of other alkali-metal atoms), and the technique of saturated absorption spectroscopy (SAS) to get narrow Doppler-free hyperfine peaks.

The standard way to solve this problem is to collect fluorescence from an atomic beam excited by a perpendicular laser beam. The perpendicularity ensures that the first-order Doppler effect is minimized; but it is not really zero (Doppler free) because of a small misalignment angle from perpendicularity and any divergence of the beam. In fact, the lineshape of each peak is not Lorentzian but Voigt (combination of Lorentzian for spontaneous decay and Gaussian for Doppler broadening). However, if one still wants an SAS spectrum with Doppler-free Lorentzian peaks, then the solution is to use an actively pumped stainless steel (SS) chamber with high-enough vapor pressure of Li to get significant absorption^2^.

In this work, we use the atomic-beam technique to measure the hyperfine interval in the 3S_1/2_ state of ^7^Li. The state is populated using a two-step laser excitation process—the first one is a diode laser at 671 nm to populate intermediate 2P_3/2_ state, while the second one is a diode laser at 813 nm that takes it to the upper 3S_1/2_ state. The motivation for the measurement is that there are two conflicting high-precision values reported in the literature—one using Stark spectroscopy of Rydberg state reported in 1995^3^, and the second using two-photon laser spectroscopy reported in 2003^4^. The value from the former is 189.36(43) MHz, while the value from the latter is 186.212(22) MHz. Our technique is different because it uses two electric dipole allowed single-photon transitions, and does not rely on locking to the second laser. Our value of 186.19(10) MHz is consistent with the more recent measurement reported in ref.^4^. This value is also in good agreement with theoretical calculations^5,6^.

## Experimental Details

**Figure 1:**
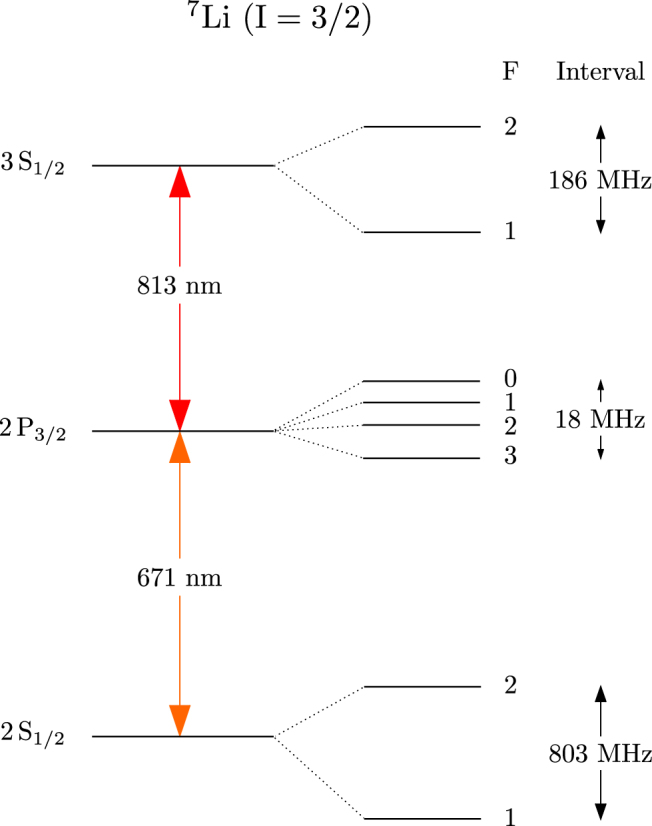
Relevant low-lying energy levels of ^7^Li (not to scale).

The relevant low-lying energy levels of Li are shown in Fig. 1. The lower transition from the ground state is the 2S_1/2_ → 2P_3/2_ transition at 671 nm. The upper transition from the intermediate state is the 2P_3/2_ → 3S_1/2_ transition at 813 nm. Both transitions are strong because they are electric dipole (E1) allowed; hence they can be driven using low-intensity laser beams.

**Figure 2:**
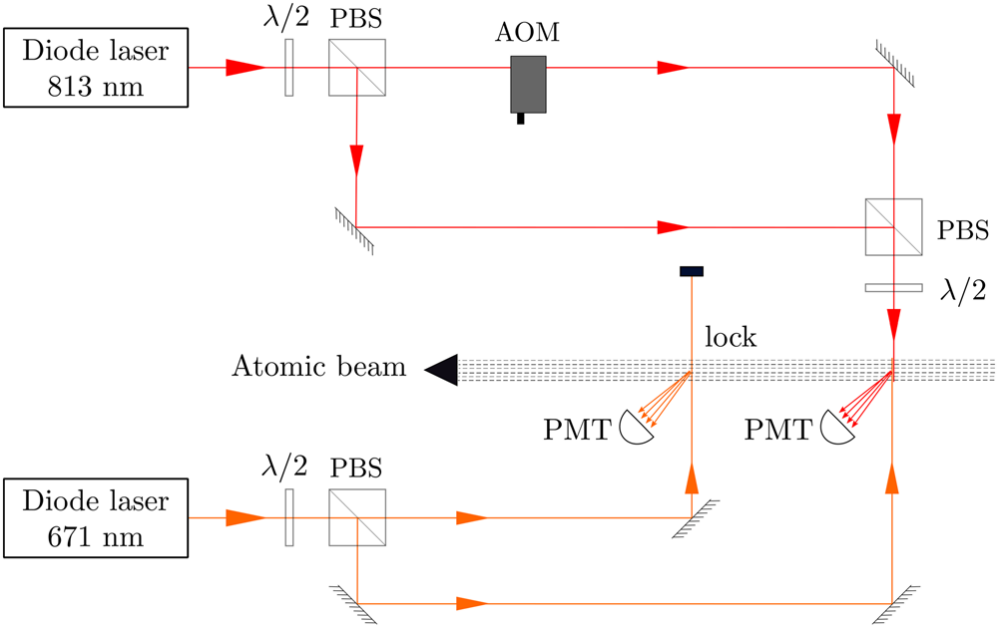
Experimental schematic. Figure key: *λ*/2 – halfwave retardation plate; PBS – polarizing beam splitter; AOM – acousto-optic modulator; PMT – photomultiplier tube.

The experimental setup is shown schematically in Fig. 2. The laser beams driving the two transitions are derived from feedback-stabilized diode laser systems, as described in ref.^7^. The one at 671 nm uses a grating with 2400 lines/mm, while the one at 813 nm uses a grating with 1800 lines/mm.

The laser beam at 671 nm has an elliptic shape, with 1/*e* ^2^ diameter of 1 mm × 4 mm. Its power is controlled using a halfwave (*λ*/2) retardation plate followed by a polarizing beam splitter cube (PBS). The laser frequency is locked using modulation at 30 kHz of the injection current into the laser diode. As seen from Fig. 1, the hyperfine levels in the D_2_ line (2S_1/2_ → 2P_3/2_ transition) are too closely spaced to be resolved completely. Therefore, the laser is locked to the unresolved F = 2 → F′ peak in the D_2_ line—the exact lock point is not important as long as transitions to the upper state are allowed. Both 1 → F′ and 2 → F′ will work; however, 2 → F′ is chosen because the peak is more prominent. This point is explained further when we discuss the experimental spectrum.

The laser beam at 813 nm also has an elliptic shape with 1/*e* ^2^ diameter of 1.5 mm × 4 mm. The measurement requires the 813 nm beam to be shifted by a known frequency. This is achieved using an acousto-optic modulator (AOM). The frequency of the AOM is in the RF range, which is set by a frequency generator (HP8656B) with a timebase accuracy of 10^−6^. Both the unshifted and AOM-shifted 813 nm beams are used for the experiment. As seen in the figure, the two beams are separated and combined using PBSs. The polarization of the combined beam is adjusted using a *λ*/2 plate. The combined beam counter-propagates with the locked 671 nm beam for the two-step excitation to the 3S_1/2_ state. The polarization of the combined 813 nm beam is adjusted to get significant heights for both unshifted and AOM-shifted beams.

All the required spectroscopy experiments are done by having an atomic beam inside an ultra-high vacuum (UHV) system, maintained at a pressure below 10^−7^ torr using a 40 l/s ion pump. The Li source consists of an SS vial containing a small ingot of unenriched Li. The vial is resistively heated to a temperature of about 200 °C. When heated, the source produces an atomic beam containing both stable isotopes of Li, namely ^6^Li and ^7^Li. The atomic beam is mechanically collimated with a divergence angle of 0.1 mrad using apertures. The pressure rises by 2 orders-of-magnitude when the source is turned on. The two laser beams intersect the atomic beam at right angles, which as mentioned before minimizes the first-order Doppler shift. The fluorescence signals are collected by photomultiplier tubes (PMTs).

## Results and Discussion

### Experimental results

**Figure 3:**
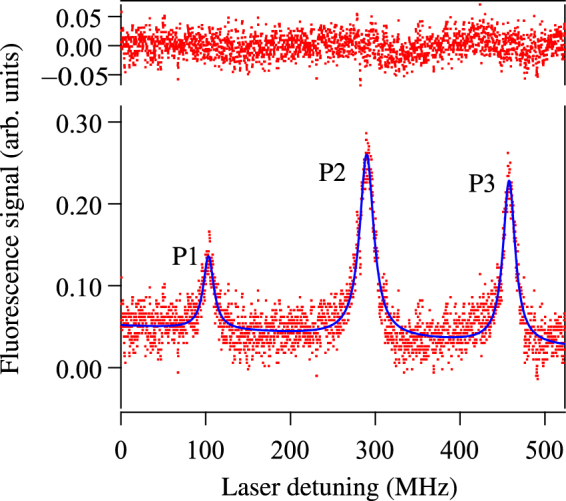
Fluorescence signal from the 3S_1/2_ → 2P_3/2_ spontaneous decay of the upper state. P1 corresponds to the *F* = 2 level; P2 corresponds to the *F* = 1 level; and P3 corresponds to the *F* = 1 level again but shifted by the AOM frequency. The solid line is a Lorentzian fit to the 3 peaks with the fit residuals shown on top.

A typical spectrum in ^7^Li used for the experiment is shown in Fig. 3. The fluorescence signal obtained from decay of the 3S_1/2_ state to the intermediate 2P_3/2_ state is plotted as a function of 813 nm laser frequency. The AOM shift for the spectrum shown is 168 MHz. The first peak (P1) corresponds to the *F* = 2 level of the 3S_1/2_ state; the second peak (P2) corresponds to the *F* = 1 level of the 3S_1/2_ state; and the third peak (P3) is the second peak along with the AOM shift. The solid line is a multipeak fit to the 3 peaks with a Lorentzian lineshape for each peak. Even though, as mentioned in the introduction, the lineshape of each peak is a Voigt function, we have used a Lorentzian function because it fits the data quite well, as seen from the featureless residuals on top of each peak (there is some structure in the region between peaks P2 and P3, which arises because of electronics noise in our PMT). In addition, the error (which depends on the signal-to-noise ratio) is well-defined for a Lorentzian function.

The linewidth of each peak is 15–20 MHz, which is larger than the expected linewidth of the 3*S*_1/2_ → 2*P*_3/2_ transition—a combination of the 5.25 MHz natural linewidth of the 3*S*_1/2_ state^8^ convolved with the 6 MHz natural linewidth of the 2P_3/2_ state. The increase in linewidth arises for the following reasons.

(i) Small misalignment angle from perpendicularity of the laser and atomic beams—we know that the angle is small because the increase in linewidth is quite small.
(ii) Residual divergence of the atomic beam.
(iii) Closely spaced (less than the natural linewidth) hyperfine levels of the 2P_3/2_ state.

The *x* axis in Fig. 3 is scaled with the known AOM shift between peaks P1 and P3. The axis is nonlinear because it varies as the sine of the grating angle, but is close to linear on this scale. This also shows why the exact lock point of the 671 nm laser is unimportant. A change in the lock point will cause the zero point in the figure to shift, but the separation between P1 and P3 will not change; thus the scaling of the laser scan axis will not be affected.

Since the hyperfine interval to be measured is near 190 MHz, the AOM shift is varied from 160 to 212 MHz, in steps of 2 MHz. At each value of AOM shift, a spectrum of the kind shown in Fig. 3 is recorded. A multipeak fit with Lorentzian lineshape for the 3 peaks yields each peak’s location and error in the location. The hyperfine separation (HFS) is the difference in location between peaks P1 and P2, while the separation between peaks P2 and P3 is the AOM frequency. The quantity

1 $$\begin{array}{rcl}\delta  & = & {\rm{HFS}}-{\rm{AOM}}\\  & = & (\mathrm{P3}-{\rm{P1}})-2\,{\rm{AOM}}\end{array}$$

has a zero crossing when the AOM frequency is equal to the HFS. The above expression shows that the error in *δ* is equal to the sum of the errors in P1 and P3.

**Figure 4:**
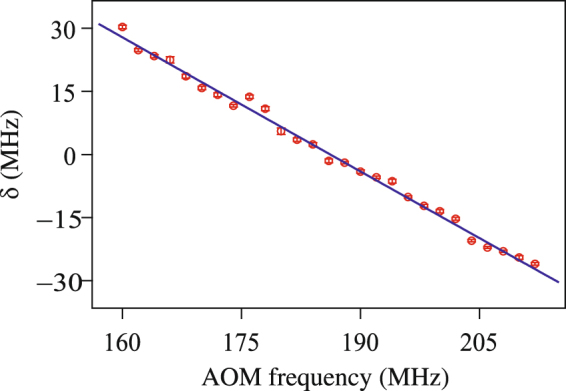
Peak separation *δ* (as defined in the text) plotted as a function of AOM frequency. The solid line is a weighted second-order polynomial fit, weighted by the error bar for each point. The error bar for each point is smaller than the symbol, and not seen clearly.

The quantity *δ* as a function of AOM frequency is shown in Fig. 4. Each value also has an error bar as determined above. The solid line is a weighted polynomial fit, weighted by the error bar for each point. Since the laser scan axis is nonlinear, the order for the polynomial fit is chosen to be the minimum nonlinear one (namely second order) — first order (or linear) gives a ten times larger error. We have also verified that the zero crossing of the fit remains unchanged within its error when we use higher-order polynomials. The zero crossing of the fit along with its error yields the HFS as 186.19(10) MHz. Since the hyperfine interval is related to the hyperfine constant as 2*A*, the measured value of the constant is *A* = 93.095 ± 0.050 MHz, where the error is the statistical error in the curve fit.

Scanning the laser to get the entire spectrum has many advantages compared to the other technique that we have developed where the AOM frequency is locked to a hyperfine peak^9^. The main advantage is that the technique avoids servo-loop errors, as demonstrated by us in ref.^10^. Another advantage is that the measured interval is independent of scaling of the laser scan axis. Any such rescaling will change the *y*-axis of Fig. 4, but not the zero crossing.

### Error analysis

The different sources error in the measurement are listed below. The values for each one are detailed in the attached supplementary file.

1. Error in the curve fit – 50 kHz.
2. Doppler shift error due to non-overlap of shifted and unshifted 813 nm beams – 10 kHz.
3. AC Stark shift, which causes the lineshape to deviate from Lorentzian – 5 kHz.
4. Optical pumping into Zeeman (magnetic) sublevels in the presence of stray magnetic fields – 10 kHz.
5. Velocity redistribution due to radiation pressure – 5 kHz.
6. Collisional shift – 1 kHz.
7. AOM frequency timebase error – 0.3 kHz.

Adding the above sources of error in quadrature yields the final error in the measurement as 52.45 kHz. Thus the value of the hyperfine constant measured in this work is

$$A=93.095\pm 0.052\,{\rm{MHz}}$$

### Comparison to previous values

**Figure 5:**
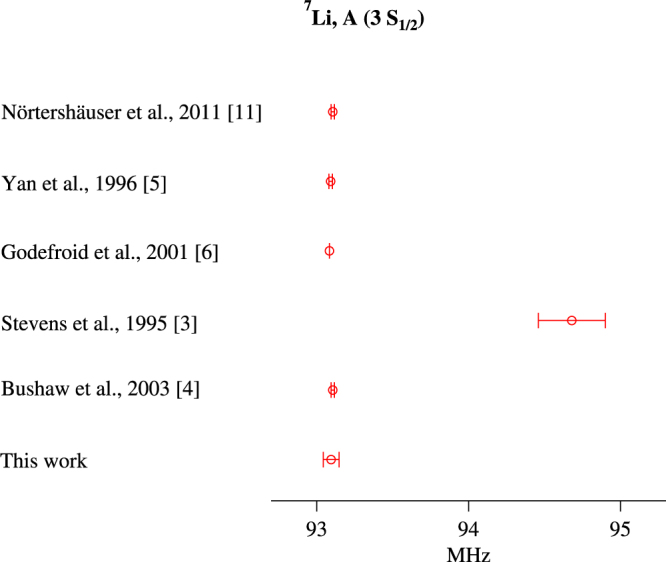
Hyperfine constant *A* in the 3S_1/2_ state of ^7^Li measured in this work compared to earlier work.

Our present measurement is compared to the two conflicting earlier experimental values in Fig. 5. It is clear that our value is consistent with the value of Bushaw *et al*.^4^, but quite inconsistent with the value of Stevens *et al*.^3^. This shows that the work in ref.^3^ may have been plagued by unaccounted systematic errors. The figure also shows that our value is consistent with theoretical calculations, done with Hylleraas variational method^5^ and multiconfiguration Hartee-Fock method^6^. We also show that our value compares favorably with results of isotope-shift measurements in Li (both stable and short lived) done at GSI in Germany^11^. The authors extract the value of the relevant hyperfine constant using a combination of theory and experiment.

## Conclusions

In summary, we have measured the hyperfine constant in the 3S_1/2_ state of ^7^Li. The state is populated using two single-photon transitions via the intermediate 2P_3/2_ state. Both transitions are excited using diode lasers. This method is different from previous techniques used in refs^3,4^, which report discrepant values for the hyperfine constant. Our value of *A* = 93.095(52) MHz is consistent with the more recent measurement in ref.^4^, which uses two-photon spectroscopy for excitation from the ground state. Our value is also consistent with theoretical calculations.

## Electronic supplementary material

Supplementary information
