# Supplementary material for: Precise measurement of hyperfine structure in the 3S1/2 state of 7Li
Source: Sci Rep. 2017 Oct 16;7:13204. doi: 10.1038/s41598-017-13531-9 (PMC5643324; doi:10.1038/s41598-017-13531-9)
Supplement: Supplementary file 1 — Supplementary information [file 41598_2017_13531_MOESM1_ESM.pdf]

Precise measurement of hyperfine structure in the $3\,S_{1/2}$ state of ^{7}Li

Pushpander Kumar[1] and Vasant Natarajan[1,*]

[1]Department of Physics, Indian Institute of Science, Bangalore-560012, India
[*]vasant@physics.iisc.ernet.in

Supplementary information

1. **Curve fitting error**. Obtained from the statistical error from the curve fitting algorithm.

2. **Doppler shift error due to non-overlapping 813 nm beams**. This error is estimated by deliberately misaligning the beams by a large angle and measuring the shift, and using the misalignment angle of 1 μrad in the experiment.

3. **AC Stark shift**. The AC Stark shift arises due to nearby hyperfine levels from which the laser is detuned. Its effect is to cause the lineshape of the peak to deviate from Lorentzian. Since the error is power dependent, it is estimated by repeating the measurement at different powers.

4. **Zeeman error**. The primary effect of a magnetic field is to split the Zeeman sublevels and broaden the line without affecting the line center. However, line shifts can occur if there is asymmetric optical pumping into Zeeman sublevels. For a transition $|F, m_F\rangle \rightarrow |F', m_{F'}\rangle$, the systematic shift of the line center is $\mu_B(g_{F'}m_{F'} - g_F m_F)B$, where $\mu_B = 1.4$ MHz/G, is the Bohr magneton, g's denote the Landé g factors of the two F levels, and B is the magnetic field. The selection rules for dipole transitions are $\Delta m = 0, \pm 1$, depending on the direction of the magnetic field and the polarization of the light. Thus, if the beams are linearly polarized (ensured by the use of PBS's), there will be no asymmetric pumping and the line center will not be shifted. However, there could be some circularity in the polarization introduced by the birefringence in the glass cell. The error is estimated using 1% of circularity in the presence of a stray magnetic field of 10 mG (measured using a three-axis fluxgate magnetometer).

5. **Radiation pressure error**. Radiation pressure causes velocity redistribution of the atom in the beam. This will again show up as a non-Gaussian component of the lineshape. Therefore, the size of the error is conservatively estimated from the deviation from a Gaussian profile.

6. **Collisional error**. To first order, collisional shifts are the same for different hyperfine levels, and hence do not affect the interval. Small differential shifts of the interval have been studied carefully in the ground state of Cs, due to its importance in atomic clocks. However, the size of the shift (in a vapor cell) is in the mHz range [1]. We expect the shift in the excited state to be of the same range. In addition, the shift in an atomic beam will be a factor of 10 smaller. Therefore, we conservatively estimate the error to be 1 kHz.

7. **AOM frequency error**. Obtained from the timebase error of 10^{-6} multiplied with the maximum AOM frequency of 300 MHz (which is slightly larger than the one used in the experiment).

References

[1] F. Pereira Dos Santos, H. Marion, S. Bize, Y. Sortais, A. Clairon, and C. Salomon. Controlling the cold collision shift in high precision atomic interferometry. *Phys. Rev. Lett.*, 89:233004, 2002.
